# Congenital Diaphragmatic Hernia and Congenital Nephrotic Syndrome in a Low-Birth-Weight Infant: A Case Report

**DOI:** 10.1155/crpe/3135018

**Published:** 2025-10-17

**Authors:** Yotaro Misaki, Ryo Oi, Taiki Haga, Takahiro Ishida, Takaaki Sakaguchi, Takuya Matsuda, Takao Kazuta, Toshiaki Kan, Hikoaki Oba, Yoko Akamine, Ryutaro Hisatomi, Rika Fujimaru, Yuichi Takama, Takashi Sasaki, Yasuyoshi Otsuka

**Affiliations:** ^1^Department of Pediatric Critical Care Medicine, Osaka City General Hospital, Osaka, Japan; ^2^Department of Pediatric Nephrology, Osaka City General Hospital, Osaka, Japan; ^3^Department of Pediatric Surgery, Osaka City General Hospital, Osaka, Japan

## Abstract

We encountered a case of congenital diaphragmatic hernia (CDH) and congenital nephrotic syndrome (CNS) in a low-birth-weight infant weighing < 2 kg. Dialysis was required due to progressive acute kidney injury in the early postnatal period, and a peritoneal dialysis (PD) catheter was placed during CDH repair surgery. During the postoperative acute phase, continuous hemodialysis (CHD) was performed to minimize stress on the surgical site; however, owing to PD-related peritonitis and sutural insufficiency of the diaphragm, the transition to PD was not feasible, necessitating prolonged CHD. During the course of the illness, the patient developed vascular occlusion in the vessels suitable for blood access and superior vena cava syndrome, ultimately rendering continued CHD and PD impossible, leading to death at 74 days of age. Respiratory and circulatory management are required during the perioperative period of CDH repair, necessitating multiple supportive therapies and careful nutritional management. This leads to a complex vicious cycle of complications, including protein loss due to CNS, coagulation disorders, circulatory failure, delayed wound healing because of malnutrition, increased susceptibility to infection, thrombosis, and infections associated with CHD and PD. Optimization of supportive therapies, including systemic anticoagulation management, infection control, and early establishment of nutrition, is considered crucial for the safe implementation of CHD or PD in such diseases.

## 1. Introduction

Congenital diaphragmatic hernia (CDH) and congenital nephrotic syndrome (CNS) are rare conditions, and cases of their coexistence are even rarer, with only a few reported cases [1] and no established treatment protocols. CDH occurs when the abdominal organs herniate into the thoracic cavity during fetal development, leading to lung hypoplasia and respiratory and circulatory dysfunction due to persistent pulmonary hypertension in the neonatal period. Intensive care and hernia repair surgery are required during the neonatal period [2]. Additionally, CNS is associated with severe proteinuria and hypoalbuminemia, which can lead to circulatory failure and thrombosis due to coagulation disorders. As renal failure progresses, renal replacement therapy (RRT), such as hemodialysis or peritoneal dialysis (PD), may be required [3]. Although several gene mutations have been implicated in CNS, and CDH has been linked to various syndromes, only a few case reports describe patients presenting with both conditions simultaneously [1, 2, 4], and little is known about the best management strategies for such complex scenarios. This represents a significant clinical gap, as the coexistence of these two conditions leads to compounded complications involving respiratory failure, fluid balance, nutritional management, infection risk, and vascular access for RRT. In this report, we present a case of an infant with low birth weight who required hernia repair surgery and multidisciplinary treatment, including RRT, during the neonatal period, but could not be saved. To the best of our knowledge, this is one of the few reported cases of coexisting CDH and CNS in a neonate, highlighting the complexity of neonatal intensive care management in such cases. We aimed to contribute to the limited body of literature on this topic and highlight the need for further research and case accumulation to inform future guidelines.

## 2. Case Presentation

The patient was a female infant aged 0 days, with a birth length of 46 cm and a birth weight of 1890 g. The mother was a 33-year-old gravida 2 para 1 woman. The infant was diagnosed with CDH in utero and delivered via emergency cesarean section at 36 weeks and 0 days of gestation because of poor fetal head descent following the onset of labor. The Apgar score was two at 1 min and five at 5 min. Endotracheal intubation was performed immediately after birth, and airway management with high-frequency oscillatory ventilation (HFOV) and inhaled nitric oxide (iNO) therapy was initiated shortly thereafter. After the administration of sedatives (midazolam and fentanyl citrate) and muscle relaxants (rocuronium bromide), the patient was admitted to the pediatric intensive care unit (PICU).

Upon admission to the PICU, the patient presented with a heart rate of 140 bpm, a blood pressure of 40/30 mmHg, and SpO_2_ levels of 97% (right upper limb) and 92% (lower limb), demonstrating a significant difference in SpO_2_ between the upper and lower limbs, as well as hypotension. Physical examination revealed a flat fontanelle, clear bilateral lung sounds on artificial ventilation, no heart murmurs, a flat and soft abdomen, and no other external abnormalities.

**Figure 1:**
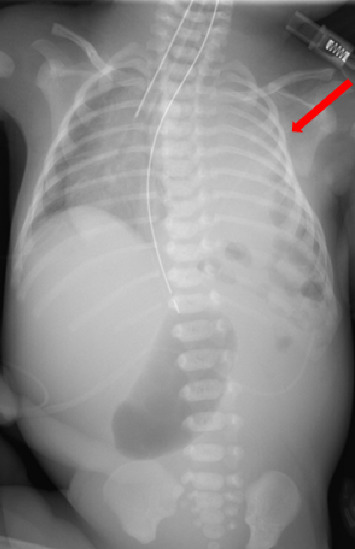
Chest X-ray image taken when entering PICU. The intestinal tract has prolapsed into the thoracic cavity, and the permeability of the left lung field has decreased.

Admission laboratory findings (Table 1) showed a blood urea nitrogen of 10.6 mg/dL and a creatinine level of 0.78 mg/dL, with no evidence of renal dysfunction [5], and an albumin level of 0.5 g/dL, which was low; therefore, 5% albumin solution was administered. Chest radiography (Figure 1) revealed intestinal gas shadows in the left thoracic cavity, a decreased left lung field, and a mediastinal shift to the right. Transthoracic echocardiography revealed good ventricular contractility, patent ductus arteriosus with a bidirectional shunt, patent foramen ovale, and no other cardiac anomalies.

A central venous catheter (CVC) was inserted into the right femoral vein, and intravenous administration of epinephrine, alprostadil alfadex, muscle relaxants (rocuronium bromide), and sedatives (midazolam and fentanyl citrate) was initiated, with management continuing until hernia repair surgery. Additionally, during CVC use, 2 U/kg/h dalteparin sodium was administered for systemic anticoagulation therapy. Respiratory and circulatory parameters were stabilized, allowing a reduction in FiO_2_. However, by 3 days of age, a gradual decrease in urine output and the progression of renal dysfunction prompted further investigations. Acute kidney injury (AKI), severe proteinuria, hypoalbuminemia, generalized edema, preexisting fetal ascites, and a massive placenta weighing > 25% of the infant's birth weight (555 g, approximately 29% of the infant's birth weight), along with elevated maternal *α*-fetoprotein at 755 ng/mL, led to the diagnosis of CNS [3]. Subsequent genetic testing confirmed the presence of a missense mutation c.1316G > *A*, p.Arg439His in exon 8 of the *WT1* gene.

**Figure 2:**
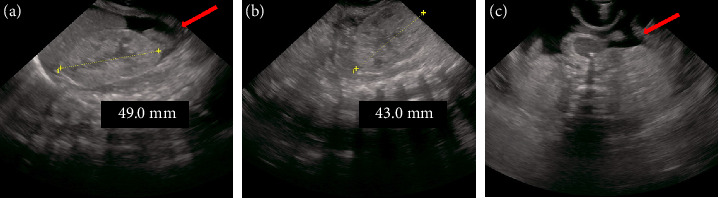
Abdominal ultrasound image at the time of diagnosis of CNS. No difference exists in the size of the kidneys ((a) right kidney, (b) left kidney). No hypoplasia or cystic kidney was observed. Ascitic fluid was observed (a, c).

**Figure 3:**
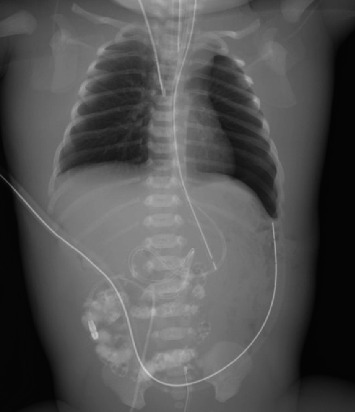
After CDH repair surgery, a PD catheter was placed in the abdominal cavity.

Abdominal ultrasound (Figure 2) revealed ascites, a right kidney measuring 49 mm, and a left kidney measuring 43 mm with no obvious hypoplasia. Owing to the rapid worsening of edema and lack of improvement in renal function or urine output, RRT was considered necessary. PD is necessary for long-term survival in patients with CNS. At 3 days of age, a 6 Fr blood access was secured via the right internal jugular vein, followed by CDH repair surgery and placement of a PD catheter in the abdominal cavity (Figure 3). The diaphragmatic defect, measuring 2 × 1 cm, was closed with direct sutures.

**Figure 4:**
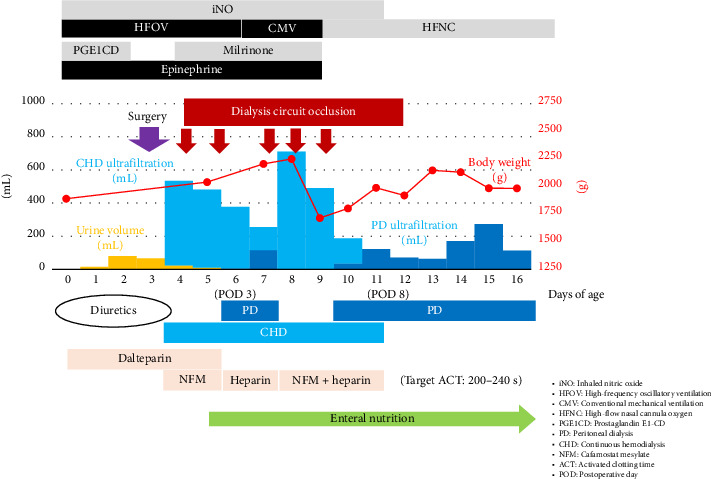
Progress chart after initial admission to PICU.

Postoperatively (Figure 4), HFOV was continued, and conventional mechanical ventilation was initiated on postoperative day (POD) 3. After extubation on POD 6, treatment was continued using a high-flow nasal cannula. Epinephrine and milrinone, which were used for postoperative cardiac support, were discontinued on POD 6, and iNO was discontinued on POD 8. Supportive therapies included sedatives (dexmedetomidine hydrochloride, midazolam, and fentanyl citrate), antibiotics (ampicillin sodium and sulbactam sodium), and blood transfusions. Regarding nutritional management, parenteral nutrition was administered in the early postoperative period, enteral nutrition with regular milk was initiated on POD 2, and enteral nutrition alone was continued from POD 4. Meanwhile, regarding the CNS, urine output decreased postoperatively and became anuric from POD 3 onward. Continuous hemodialysis (CHD) was initiated approximately 4 hours postoperatively. Blood priming with concentrated red blood cells was performed to prevent hypotension at the start of dialysis, and in-circuit dialysis was performed before connecting the patient to the circuit. The CHD settings were initially as follows: blood flow rate (Qb), 20 mL/min, and dialysate flow rate (Qd), 100 mL/h. Subsequent adjustments were based on the patient's condition, with Qb ranging from 15 to 20 mL/min and Qd ranging from 50 to 100 mL/h. Regarding the anticoagulants used during CHD, we initially started with nafamostat mesylate alone. However, due to circuit occlusion occurring within a short time, we switched to heparin sodium alone. However, circuit occlusion continued to occur within a short period. Therefore, we switched to a combination of nafamostat mesylate and heparin sodium and discontinued dalteparin sodium. Anticoagulation monitoring was performed using a Hemochron Signature Elite (International Technidyne Corporation, Edison, NJ, USA), with the target activated clotting time (ACT) set at 200–240 s. Nafamostat mesylate was administered at a dose of 0.5–1.7 mg/kg/h, and heparin sodium was adjusted within the range of 10–13.5 U/kg/h. During this period, including the period when the ACT target value was achieved, five dialysis circuit occlusions occurred, all occurring relatively early, within 3–18 h. PD was initiated on POD 3, and the volume of fluid removed by PD increased from POD 4. CHD was discontinued on POD 8, and because the patient's general condition stabilized after switching to PD alone, the patient was transferred to the neonatal intensive care unit (NICU) on POD 13 at 16 days of age.

**Figure 5:**
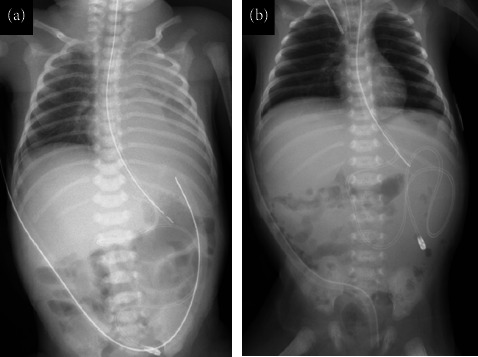
The communication between the thoracic cavity and the abdominal cavity was found due to the accumulation of chylous pleural effusion (a). The diaphragm was repaired again and a PD catheter was inserted (b).

In the NICU, the patient developed PD-related peritonitis caused by *Methicillin-Resistant Staphylococcus epidermidis* at 21 days of age, and treatment with intraperitoneal vancomycin (VCM) was initiated. Poor PD drainage and left pleural effusion were observed. At 44 days of age, PD drainage became chylous, raising suspicion of a communication between the thoracic and abdominal cavities, which made PD use difficult. At 45 days of age, laparoscopic diaphragmatic repair and PD catheter replacement were performed (Figure 5), and the patient was readmitted to the PICU for postoperative management. A slit-like defect was discovered at the central suture site of the initial repair, which was closed using direct suturing.

**Figure 6:**
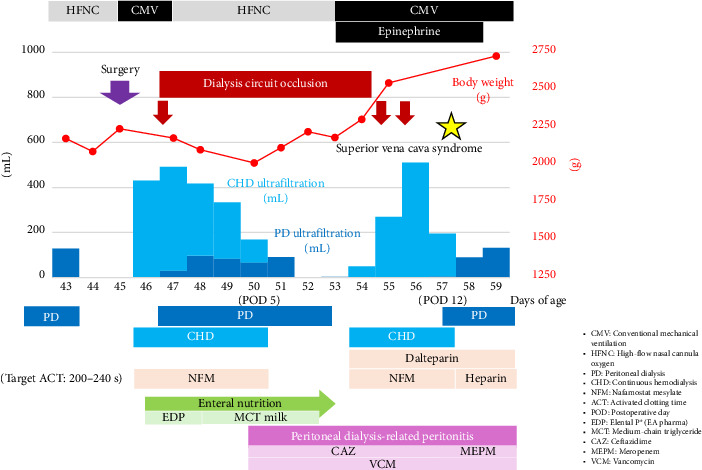
Progress chart after readmission to the PICU following diaphragmatic repair surgery.

The postoperative course in the PICU after reoperation (Figure 6) was similar to that during the initial admission. Early postoperative management included CHD initiation, and anticoagulation therapy was adjusted with nafamostat mesylate at 0.5–1.2 mg/kg/h to achieve an ACT of 200–240 s. PD was resumed after extubation on POD 2. CHD was discontinued on POD 5, and the respiratory and circulatory statuses were stable. However, recurrence of PD-related peritonitis caused by *Klebsiella oxytoca* occurred following turbidity in the PD effluent, and intraperitoneal administration of ceftazidime and VCM was initiated. As the volume of PD effluent decreased, fluid removal via PD alone became difficult. On POD 8, leakage of the dialysate from the PD catheter exit site to the skin surface was detected, and CHD was reintroduced. The left internal jugular vein and both femoral veins were too narrow for catheter placement. Therefore, a blood access catheter was inserted into the right internal jugular vein. Thrombosis was observed within the right internal jugular vein, and adequate blood withdrawal was impossible unless the catheter tip was positioned within the right atrium, necessitating continued CHD for life support. However, frequent circuit stoppages and occlusions due to increased pressure in the CHD circuit made adequate fluid removal impossible. Additionally, significant worsening of facial edema occurred on POD 12, leading to a diagnosis of superior vena cava syndrome (SVCS) secondary to blood access insertion and venous thrombosis. Continuation of CHD was deemed difficult thereafter, and PD alone was resumed; however, persistent leakage to the body surface made continued PD management challenging. On POD 15, at 60 days of age, the patient was transferred to the NICU, where the PD catheter exit site was sutured; however, the improvement was minimal. Subsequently, fluid management using PD became impossible. At 68 days of age, PD was discontinued, and palliative care was initiated. The patient died at the age of 74 days.

## 3. Discussion

In this case, the pathophysiology of CDH and CNS, combined with the physical limitations imposed by a low birth weight of < 2 kg, resulted in highly complex and challenging intensive care management. CDH is a congenital malformation characterized by incomplete closure of the diaphragm and herniation of fetal abdominal organs into the thoracic cavity, leading to pulmonary hypoplasia, pulmonary hypertension due to vascular remodeling, and cardiac dysfunction [2]. CNS typically presents within the first 3 months of life, with podocyte gene abnormalities as the primary cause. In rare cases, it may result from congenital infections or maternal autoimmune diseases. Owing to severe proteinuria and hypoalbuminemia, CNS can lead to impaired hemodynamics, increased susceptibility to infection, thrombosis caused by reduced circulating blood volume and loss of anticoagulant factors, growth disorders involving multiple factors, and progressive renal failure, among other complications [3]. More than 70 syndromes associated with CDH have been reported, including *WT1* syndrome; however, no single genetic cause accounting for > 3% of CDH cases has been documented [2]. Multiple gene mutations have been reported to cause CNS. The *WT1* mutation, which exhibits a wide range of phenotypes, leads to end-stage renal failure in early infancy [1, 6]. The association between CDH and CNS is uncommon, with only a few reported cases, including a case of idiopathic nephrotic syndrome combined with esophageal diaphragmatic hernia reported by Galloway and Mowat in 1968 [4], and a report of nephrotic syndrome with *WT1* mutations, in which diaphragmatic defects or hernias were rarely associated with each other [1].

Various clinical challenges have been identified, as follows.

### 3.1. Challenges in Renal Replacement Therapy

Generally, as a long-term treatment strategy for CNS, PD is initiated at 6–12 months of age, when a certain weight and nutritional status are expected, targeting a body weight of approximately 10 kg for kidney transplantation [3]. In this case, performing PD before CDH repair surgery was not feasible due to dialysate leakage into the thoracic cavity through the diaphragmatic defect, concerns about postoperative suture failure, and the necessity of initiating hemodialysis prior to PD. The factors contributing to the rapid progression of AKI and anuria in the early neonatal period included circulatory failure due to CDH, medication use for CDH management, such as catecholamines and prostaglandin agents, and reduced renal blood flow resulting from the maintenance of a patent ductus arteriosus [7]. Additionally, the rapid progression of renal failure associated with *WT1* mutations may have contributed to this case. The primary challenges in managing both CDH and CNS are implementing RRT and managing complications that worsen with RRT. Hemodialysis in neonates weighing < 2 kg has rarely been reported [8], and our institution has limited experience in this regard. In the early postoperative period following CDH surgery, concerns about infection and CDH recurrence exist; therefore, delaying the initiation of PD as much as possible was necessary. Ideally, PD should be initiated after allowing the peritoneum to rest; however, owing to anuria and end-stage renal failure, RRT was required immediately postoperatively. We initiated CHD with a Qb of 20 mL/min through the right internal jugular vein. However, frequent circuit stoppages due to inadequate blood withdrawal compromised stable management. Additionally, circulatory instability caused by intravascular dehydration, low albumin levels in the CNS, and a hypercoagulable state are believed to have contributed to early circuit occlusion on multiple occasions. During CVC insertion, dalteparin sodium was administered systemically, and during CHD, nafamostat mesylate and heparin sodium were administered as anticoagulants. However, following a diagnosis of CNS or if a thrombus is observed, more rigorous systemic anticoagulation management is desirable.

### 3.2. The Dilemma of Vascular Access

In CDH, approximately 80% of affected infants undergo repair surgery within the first week of life [2]. Postnatal intensive care management and supportive therapy, including measures to prevent lung injury, reduce pulmonary vascular resistance, and support cardiac function, are critical. Therefore, securing central venous access is essential. In CNS, the loss of anticoagulant factors (AT-III, protein C, and protein S) and the placement of a CVC increase the risk of thrombosis. Therefore, unnecessary CVC placement should be avoided to prevent both infection and thrombosis [3]. Prophylactic anticoagulant therapy with vitamin K, heparin sodium, or AT-III agents prior to CVC insertion is also an option. An appropriate blood access site is necessary to ensure adequate blood flow. Based on the patient's body size, the internal jugular veins on both sides were selected as the first choice. Generally, in newborns weighing < 2 kg, the diameters of the internal jugular and femoral veins are approximately 3 mm and 2 mm, respectively [9]. The femoral vein was deemed inadequate for sufficient blood flow, and the subclavian vein was excluded due to the risk of pneumothorax, which can be life-threatening in CDH management. At our institution, we use a 6 Fr blood access catheter for neonatal hemodialysis. In this case, vascular occlusion ultimately made it challenging to maintain blood access, and SVCS developed. During neonatal hemodialysis, adequate blood flow can be achieved even with a 4 Fr or 5 Fr catheter when using appropriate pediatric circuits [10–13]. In this case, which involved multiple hemodialysis sessions and a smaller body size, using a catheter smaller than usual, depending on the vascular diameter, might have been an option. Regarding blood access and the selection of CVC, umbilical artery–vena cava catheters are also an option for newborns; however, they are not suitable for prolonged use and may not be feasible in cases beyond 4-5 days of postnatal age [14]. Therefore, they were only usable for an extremely short perioperative period in this case. Coagulation disorders caused by underlying diseases and vascular complications resulting from prolonged CHD contributed to the development of SVCS, rendering continued CHD unachievable. In addition to the interaction between CDH and CNS, a birth weight < 2 kg further complicated treatment.

### 3.3. Nutritional and Infectious Complications

In CDH, delayed initiation of nutrition due to various factors, such as edema, ileus, and infection, as well as increased metabolism resulting from respiratory distress and growth disorders caused by gastroesophageal reflux, are also of concern [2]. Additionally, careful attention should be given to infection control strategies. Nutritional support must be initiated and escalated cautiously because of the postoperative status of CDH repair and the intestinal edema caused by CNS. The development of chylothorax during treatment necessitated a change in the nutritional formulation, which could potentially result in inadequate nutrition. Owing to the anticipated increased susceptibility to infection due to malnutrition and CNS-induced globulin loss, as well as the need to perform PD without a rest period despite the risk of postoperative wound dehiscence or peritonitis due to anuria, this was a high-risk case for PD-related peritonitis [15]. Additionally, the presence of multiple devices, such as blood access devices and CVCs, poses a high risk of infection. A dilemma existed between shortening the period of hemodialysis with an unstable hemodynamic status as much as possible and delaying the initiation of PD as much as possible to allow for peritoneal rest and wound healing at the diaphragmatic repair site. PD initiated within 14 days of catheter insertion is considered a risk factor for early peritonitis [15], and in this case, early PD-related peritonitis and diaphragmatic redefect were observed, suggesting that PD initiated on POD 3 after the first surgery should have been delayed. Additionally, if an adequate volume of dialysate cannot be administered, the required fluid removal volume cannot be achieved. However, the high volume of dialysate administered per session might have caused leakage of dialysate from the body surface. Furthermore, increasing the glucose concentration in the dialysate to ensure adequate fluid removal may contribute to the development of peritonitis associated with PD. Postoperative malnutrition and increased susceptibility to infection lead to the development of PD-related peritonitis, delayed wound healing, and sutural insufficiency of the diaphragm. Physical constraints, such as leakage of the dialysate onto the body surface, hinder the continuation of PD.

In this case, the rare coexistence of CDH and CNS has revealed several valuable clinical pearls, as follows:

• The importance of early multidisciplinary planning for RRT and vascular access in neonates with co-occurring surgical and renal emergencies:
  In cases requiring advanced respiratory and circulatory management for CDH, particularly those progressing to end-stage renal failure from the neonatal period and requiring RRT, a thorough discussion is necessary regarding several issues. If the goal is long-term survival, determining how to use RRT to bridge the period until kidney transplantation while also aiming for weight gain is crucial.
• The potential contraindications of PD after major abdominal surgery in low-birth-weight infants necessitate CHD:
  PD may be contraindicated or of high risk after major abdominal surgery, forcing reliance on CHD, which presents unique challenges in low-birth-weight infants. Issues similar to those encountered herein are anticipated in cases where PD cannot be performed because of abdominal surgery, even in patients without CDH.
• The risk of cascading complications such as thrombosis, infection, and malnutrition, highlighting the need for proactive management:
  The cascade of complications can quickly become irreversible in this population, underscoring the need for aggressive and proactive management. We had to be particularly mindful of complications related to infection control, thrombosis and anticoagulant therapy, and nutrition management.
• Genetic findings:
  In this case, a *WT1* mutation was detected, and the mutation type was c.1316G > *A*, p.Arg439his in exon 8 of the *WT1* gene. Mutations in the *WT1* gene are associated with renal and genital developmental abnormalities as well as renal tumors, and they also affect the formation of the diaphragm, in rare cases.

This case was a rare and challenging clinical scenario. Accumulation of cases with similar pathologies is required, and we hope that the insights gained from this case will enhance future clinical management.

## Figures and Tables

**Table 1:** Laboratory parameters upon admission to PICU on 0 days of age.

Parameters	Initial data	Reference range
*Urine studies*		
Protein on dipstick	4+	Negative
Blood on dipstick	3+	Negative
Urine protein	8590	mg/dL

*Biochemistry*		
Sodium	136	138–145 mEq/L
Potassium	3.2	3.6–4.8 mEq/L
Blood urea nitrogen	10.6	8.0–20.0 mg/dL
Creatinine	0.78	0.4–0.8 mg/dL
Total protein	1.7	6.7–8.3 g/dL
Albumin	0.5	3.9–4.9 g/dL
Aspartate aminotransferase	61	8–38 U/L
Alanine transaminase	8	4–44 U/L
Lactate dehydrogenase	715	124–222 U/L
Creatinine kinase	147	43–165 U/L
C-reactive protein	0.02	< 0.3 mg/dL

*Hematology*		
White blood cell count	23.27	3.3–8.6 × 10^3^/μL
Red blood cell count	5.11	3.86–4.92 × 10^6^/μL
Hemoglobin	20	11.6–14.8 g/dL
Platelet count	12.6	158–348 × 10^4^/μL

*Arterial blood gas*		
pH	7.527	7.35–7.45
PaCo_2_	22.4	32.0–45.0 mmHg
PaO_2_	59	83.0–108.0 mmHg
HCO_3_^−^	18.5	22.0–26.0 mmol/L
Base excess	−1.1	−2.0 to 2.0 mmol/L
Blood sugar	54	70–105 mg/dL
Lactate	49	5–14 mg/dL

*Note:* Urine studies were performed on 2 days of age.

## Data Availability

All data used in the study are available in the published literature.
